# Use of Clinical Audits to Evaluate Timing of Preoperative Antimicrobials in Equine Surgery at a Veterinary Teaching Hospital

**DOI:** 10.3389/fvets.2021.630111

**Published:** 2021-03-26

**Authors:** Serena Ceriotti, Roxane Westerfeld, Alvaro G. Bonilla, Daniel S. J. Pang

**Affiliations:** Department of Clinical Sciences, University of Montreal, Saint-Hyacinthe, QC, Canada

**Keywords:** antibiotics, horse, prophylaxis, anesthesia record, perioperative, arthroscopy, laparotomy

## Abstract

Based on human surgical guidelines, intravenous antimicrobials are recommended to be administered within 60 min of surgical incision. Achieving this target in horses is reportedly challenging and influenced by hospital policies. The objectives of this study were to evaluate and improve: (1) the timing of antimicrobial administration to surgical incision (tAB-INC), (2) contributions of anesthesia pre-induction (tPRI) and surgical preparation (tPREP) periods to tAB-INC, and the (3) completeness of antimicrobial recording. Two clinical audits were conducted before and after the policy changes (patient preparation and anesthesia record keeping). tPRI, tPREP, and tAB-INC were calculated and compared for elective arthroscopies and emergency laparotomies within and between the audits. The percentage of procedures with a tAB-INC <60 min was calculated. Antimicrobial recording was classified as complete or incomplete. A median tAB-INC <60 min was achieved in laparotomies (audit 1; 45 min, audit 2; 53 min) with a shorter tPREP than arthroscopies (*p* < 0.0001, both audits). The percentage of procedures with tAB-INC <60 min, tAB-INC, tPRI, and tPREP durations did not improve between the audits. There was a positive correlation between the number of operated joints and tPREP (audit 1, *p* <0.001, *r* = 0.77; audit 2, *p* < 0.001, *r* = 0.59). Between audits, antimicrobial recording significantly improved for elective arthroscopies (82–97%, *p* = 0.008) but not emergency laparotomies (76–88%, *p* = 0.2). Clinical audits successfully quantified the impact of introduced changes and their adherence to antimicrobial prophylaxis guidelines. Antimicrobial recording was improved but further policy changes are required to achieve a tAB-INC <60 min for arthroscopies.

## Introduction

Perioperative antimicrobial prophylaxis is the administration of antimicrobials in the period of time around a surgical operation, including before the incision, with the goal of decreasing the likelihood of infection following exposure to bacteria during surgery (1). In equine patients, specific guidelines for perioperative antimicrobial prophylaxis do not exist; therefore, principles are adopted and adapted from human medicine (2, 3). These include the use of early generation broad-spectrum antimicrobials, preferably administered intravenously (2, 4). Thus, penicillin and gentamicin remain the most commonly used prophylactic antimicrobials in most equine hospitals (4, 5). Additionally, guidelines suggest administration of antimicrobials with a short half-life, such as sodium penicillin, within 60 min of surgical incision to achieve therapeutic tissue concentrations at the time of surgery (1, 2, 4–8). However, previous equine studies have shown that meeting this target is difficult and influenced by hospital policies (5, 9, 10).

Clinical audit is a tool to evaluate and improve the quality of patient care (11, 12). A clinical audit cycle often consists of four steps (“PDSA”), namely “Plan,” “Do,” “Study,” and “Act” (13, 14). After identifying a clinical problem that needs to be addressed (“Plan”), the data concerning the problem are systematically collected (“Do”) and analyzed with comparison to the available standards (“Study”). The results are used to implement change(s) (“Act”) whose benefit should be reevaluated with further audit cycles (11, 14).

No specific hospital guidelines concerning the timing of preoperative antimicrobial administration were in use at the equine hospital of the University of Montreal. A clinical audit was planned in 2017 to evaluate (1) the completeness of antimicrobial recording and (2) the timing of antimicrobial administration relative to surgical incision. These results were analyzed, and the changes were implemented with the goal of driving improvement. A second audit cycle was conducted to evaluate the impact of introduced changes. We hypothesized that the initial audit cycle would reveal failures in both antimicrobial recording and meeting the pre-established target of antimicrobial prophylaxis administration (within 60 min of surgical incision). Additionally, we hypothesized that the changes introduced following the first audit would standardize the timing of antimicrobial administration, improve the recording of preoperative antimicrobial prophylaxis, and improve the timing of antimicrobial administration to surgical incision.

## Materials and Methods

### Study Design

Two clinical audits were performed at the equine hospital of the University of Montreal, between September 2017 and May 2019, following a Plan-Do-Study-Act (PDSA) format (14).

The initial “Plan” phase was performed in September 2017 and consisted of an informal review of previous anesthesia records of horses undergoing surgery, revealing frequent incomplete recording of antimicrobial dosing information, particularly the time of administration. These findings were discussed, a consensus reached, and on October 1st, antimicrobial administration guidelines were introduced (see the “Hospital policy section” below). Data collection (“Do” phase, audit 1) was performed between October 1, 2017 and March 31, 2018. Anesthesia and surgery team members outside the study team were unaware of the planned audit, to avoid the Hawthorne effect (15). Collected data were analyzed (“Study” phase), and the results were communicated to the surgery and anesthesia services (clinicians, residents, interns, and technicians). Based on the results, further measures were adopted (“Act” phase, see the “Hospital policy section” below), introduced on September 1, 2018, and a second clinical audit (audit 2) was planned with data collection between October 1, 2018 and May 12, 2019. The results of audit 2 were compared to those from audit 1.

### Data Collection

General methods of data collection were identical for both audits.

#### Completeness of Antimicrobial Recording

All surgical procedures performed under general anesthesia were eligible for inclusion. Data collected from the anesthesia record consisted of date, clinical diagnosis, surgical procedure performed, antimicrobial prophylaxis administration items (time of administration, drug(s), dose and route of administration), time of induction of general anesthesia, and time of surgical incision. Recording of antimicrobial information on the anesthesia record was considered complete if all items were recorded, absent if no items were recorded, and partial if any item was missing (e.g., administration time).

#### Timing of Antimicrobial Administration

For the two most commonly performed surgical procedures, elective arthroscopies and emergency laparotomies, the following time periods were calculated: “pre-induction phase” (tPRI), “surgical preparation phase” (tPREP), and “antimicrobial dosing to incision time” (tAB-INC). The pre-induction phase was from the time of antimicrobial prophylaxis administration until the induction of general anesthesia (managed by the anesthesia team). The surgical preparation phase was from the time of induction until surgical incision (managed by the surgery team). The antimicrobial dosing to incision time was from the time of antimicrobial prophylaxis administration until the surgical incision (sum of pre-induction and surgical preparation phases).

#### Exclusion Criteria

For the outcome “completeness of record keeping,” all data were included as the incidence of missing information was an outcome of interest, with the exception of surgical procedures for which antimicrobials were not administered. For the outcome “timing of antimicrobial administration,” data were excluded if: (1) antimicrobials were not administered before the induction of anesthesia or were administered *via* a route other than intravenously or, (2) any or all of the following times were not recorded: antimicrobial administration, anesthetic induction, and surgical incision.

### Hospital Policy for Antimicrobial Administration

Following a short period of informal record review by the study team, concerns over preanesthetic antimicrobial recording and the timing of antimicrobial administration were discussed within the equine hospital (anesthesia and surgery clinicians) and an official hospital policy was instituted before audit 1. The equine anesthesia service was identified as responsible for the administration and recording of perioperative antimicrobials (type, route, dose, and time). To minimize tAB-INC, it was requested that antimicrobial administration occurs after the patient is prepared for anesthesia (mouth rinsing and picking out hooves) and immediately before walking the patient into the induction box. Based upon the results of audit 1, the following changes and recommendations were introduced during the month before audit 2: (1) Anesthesia charts were modified to facilitate accuracy of antimicrobial recording (specific headings added for each item; antimicrobial type, dose, route of administration, and time). Targeted training of individuals involved in the anesthesia management (interns, residents, technicians, and clinicians) was provided. (2) To optimize surgical preparation time, re-organization of the surgery staffing was performed when possible, with the aim of having at least two technicians available for surgical preparation of daytime elective arthroscopies. A brief observational study by a summer student found that the median surgical preparation time (clipping, cleaning, and scrubbing) was longer when one vs. two technicians were available per joint (17.2 vs. 12.5 min, respectively). Additionally, clipping time was shorter if there was one (median time of 8.5 min) or two (median time of 4 min) technicians/joint compared with one technician clipping two joints (median time of 12 min).

### Statistical Analysis

Antimicrobial dosing information (complete, partial, and absent) was collected from the anesthesia record, and percentages (%) calculated for all surgeries in Audits 1 and 2. For the purpose of comparison between audits, data were classified as complete or incomplete (the latter combined partial and absent data) and compared with Fisher's exact test. The analysis of timing data (tPRI, tPREP, and tAB-INC) was restricted to elective arthroscopy and emergency laparotomy procedures. The data from other surgery types are available in an electronic data repository: https://doi.org/10.7910/DVN/FAPILE. The percentage of surgeries with a tAB-INC time falling within a 60-min target window was calculated for each audit and compared with Fisher's exact test. Timing data (tPRI, tPREP, and tAB-INC) were examined for normality with the D'Agostino–Pearson test. As multiple subsets were not normally distributed, non-parametric analyses were used throughout. Data were compared between surgical groups for each audit with the Mann–Whitney test. In the arthroscopy group, the tPREP phase was also examined according to the number of joints operated and differences in the duration of the tPREP phase among these groups were compared with the Kruskal–Wallis test and Dunn's multiple comparison test. Correlation between the duration of the tPREP phase and the number of joints operated was examined with the Spearman's correlation coefficient. The tAB-INC, tPRI, and tPREP periods of audits 1 and 2 were compared with the Mann–Whitney test. Values of *p* < 0.05 were considered significant and 95% CIs for the difference between the comparison groups were calculated. Data are expressed as median (range). Statistical analyses were performed by using commercial software (GraphPad Prism software v6).

## Results

### Surgical Population and Antimicrobial Use

Data from 140 and 172 surgeries were included for antimicrobial recording in audits 1 and 2, respectively. The predominant surgery types were elective arthroscopies [audit 1; *n* = 60 (42.9%), audit 2; *n* = 62 (36.0%)] and emergency laparotomies [audit 1; *n* = 33 (23.6%), audit 2; *n* = 35 (20.3%)]. These were followed by reproductive surgeries [audit 1; *n* = 19 (13.6%), audit 2; *n* = 19 (11.0%)], other clean surgeries [audit 1; *n* = 9 (6.4%), audit 2; *n* = 42 (25.0%)], clean-contaminated surgeries [audit 1; *n* = 4 (2.9%), audit 2; *n* = 6 (3.0%)], and contaminated and infected surgeries [audit 1; *n* = 15 (10.6%), audit 2; *n* = 8 (4.7%)]. Details of surgery types are provided in an electronic data repository: https://doi.org/10.7910/DVN/FAPILE.

Antimicrobial prophylaxis was administered in 97.1% (136/140) and 98.9% (170/172) of the horses, respectively in audits 1 and 2, respectively. The route of administration was intravenous in all but two cases in audit 1 (procaine penicillin G, IM) and one case in audit 2 (procaine penicillin G, IM). Sodium penicillin G (IV) was administered in 88.6% (124/140) of cases in audit 1 and 85.5% (147/172) of cases in audit 2. In 7.1% (*n* = 10) of cases in audit 1 and 12.8% (*n* = 22) of cases in audit 2, sodium penicillin G was administered alone. In the remaining cases, sodium penicillin G was combined with gentamicin [audit 1; *n* = 109 (77.8%), audit 2; *n* = 120 (69.8%)] or enrofloxacin [audit 1; *n* = 5 (3.6%), audit 2; *n* = 5 (2.8%)]. Enrofloxacin (IV) was administered alone in one case (0.7%, audit 1). Ceftiofur (IV) was administered alone in five cases (3.6%) in audit 1 and in 19 cases (11.0%) in audit 2. In 2.9% (four cases) in audit 1 and 1.1% (two cases) in audit 2, the administration of antimicrobials immediately preoperatively was considered unnecessary as patients were already receiving antimicrobials (two wound debridement, one septic arthritis, one septic tenosynovitis, one maxillary fracture, and one ocular surgery). There was no record of antimicrobial administration (in either the anesthetic record or the patient record) in 2.9% (four cases, audit 1) and 1.7% (three cases, audit 2) despite a request to do so being submitted before anesthesia.

### Completeness of Antimicrobial Recording

**Figure 1:**
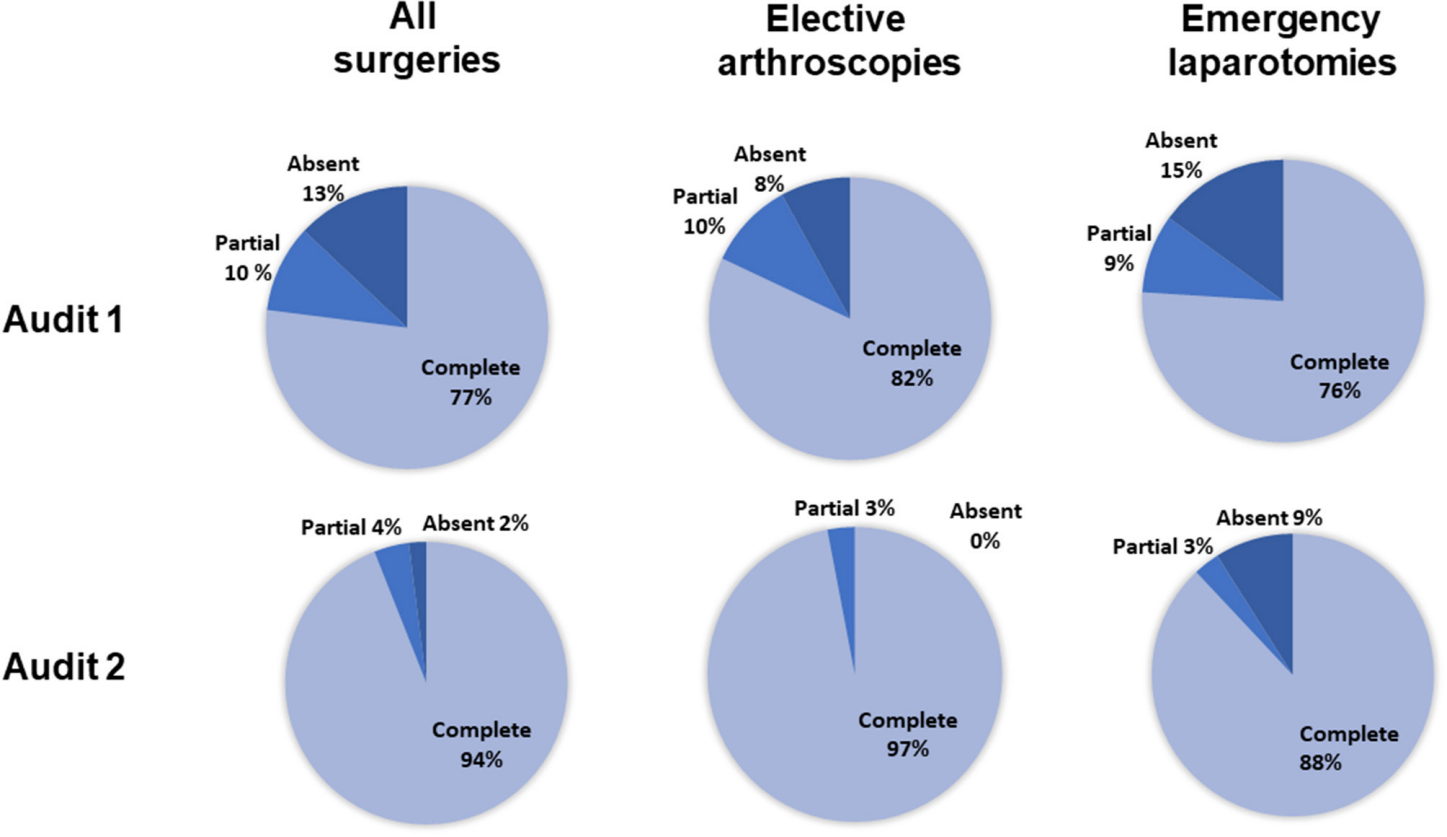
The percentage of complete, incomplete, or absent antimicrobial recordings for all surgeries, elective arthroscopies, and emergency laparotomies, in audits 1 and 2. Percentages were calculated from the total number of animals scheduled to receive preoperative antimicrobials (136 in the first audit and 170 in the second audit). For the elective arthroscopy and emergency laparotomy groups, percentages were calculated from the total number of procedures performed given that all received preoperative antimicrobials (arthroscopies: 60 first audit, 62 second audit; laparotomies: 33 first audit, 35 second audit).

For all surgeries (*n* = 312), the completeness of antimicrobial recording in the anesthetic record was significantly greater in audit 2 than in audit 1 (Figure 1, *p* < 0.001, 95% CI = 0.20–0.52). For elective arthroscopies, anesthetic record completeness was greater in audit 2 (*p* = 0.008, 95% CI = 0.03–0.70), with no significant difference between audits for emergency laparotomies (*p* = 0.2, 95% CI = 0.11–1.50).

### Timing of Antimicrobial Administration

**Figure 2:**
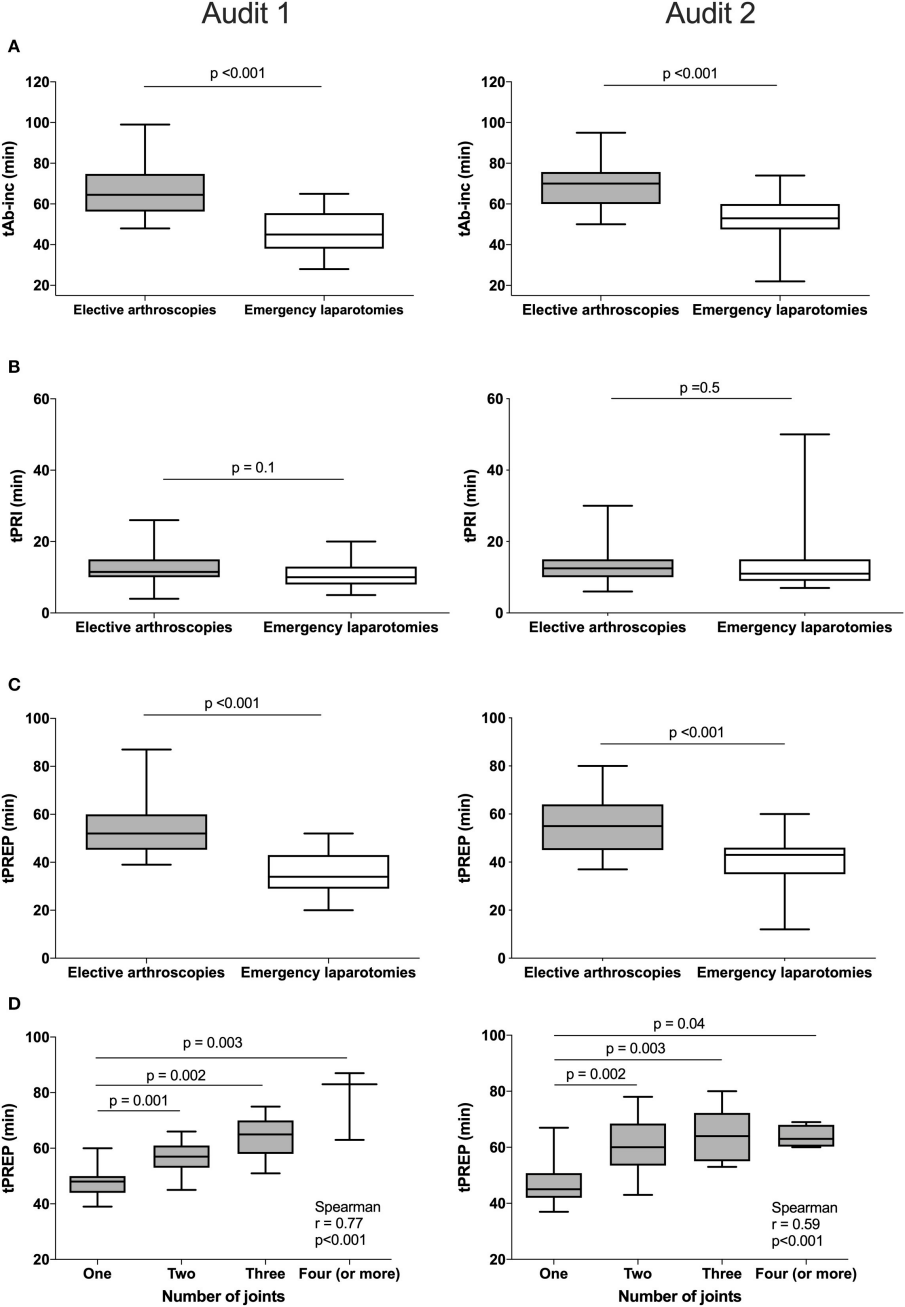
Differences in timings between elective arthroscopies and emergency laparotomies during audit 1 (left column) and audit 2 (right column) and the influence of arthroscopy joint number on preparation time. Data are expressed as median and ranges. The median antimicrobial dosing to surgical incision time (tAB-Inc) was significantly longer in elective arthroscopies than emergency laparotomies (both audits, row **A**). No significant differences in the pre-induction phase (tPRI) were detected between both the groups (both audits, row **B**), but the surgical preparation phase (tPREP) was significantly longer for elective arthroscopies (both audits, row **C**). A significant positive correlation was found between tPREP and the number of joints operated; preparation took significantly longer when more than one joint was operated but was similar between two or more joints (both audits, row **D**).

For antimicrobial timing data, 48/60 elective arthroscopies and 25/33 emergency laparotomies were eligible to be included for analysis audit 1, and 60/62 elective arthroscopies and 30/35 emergency laparotomies from audit 2. Procedures were excluded for intramuscularly administered antimicrobials (one arthroscopy, audit 1), antimicrobials administered after the induction of anesthesia (one laparotomy, audit 2), absence of antimicrobial recording (audit 1; one arthroscopy and three laparotomies, audit 2; three laparotomies), or partial recording (audit 1; six arthroscopies and three laparotomies, audit 2; two arthroscopies and one laparotomy). Similar results were found for each audit (Figure 2), with a significantly shorter tAB-INC in the emergency laparotomy group than in the elective arthroscopy group (audit 1, *p* < 0.001, 95% CI = 14.0–25.0; audit 2, *p* < 0.001, 95% CI = 11.0–21.0; Figure 2). This difference resulted from a significantly shorter tPREP for emergency laparotomies (audit 1, *p* < 0.001, 95% CI = 13.0–22.0; audit 2, *p* < 0.001, 95% CI = 10.0–20.0 min; Figure 2). In contrast, tPRI was consistent among the surgery groups (audit 1, *p* = 0.16, 95% CI = 0–3.0 min; audit 2, *p* = 0.60, 95% CI = −1.0–2 min; Figure 2). The tPREP for elective arthroscopies was positively correlated with the number of joints being prepared (audit 1, *p* < 0.0001, *r* = 0.77; audit 2, *p* < 0.0001, *r* = 0.59; Figure 2). Preparation time was significantly longer for arthroscopies when more than one joint needed to be prepared (audit 1; *p* < 0.001, audit 2; *p* < 0.001, Figure 2), although no statistical differences were found between preparing two, three, four, or more joints. In both audits, the median tAB-INC was within the recommended interval of 60 min for elective procedures involving only one joint (Table 1). For the subgroup of single joint arthroscopies, the percentage with a tAB-INC <60 min was 72 and 58% in audits 1 and 2, respectively. The percentage with a tAB-INC <60 min for the subgroup of two joint arthroscopies was 0 and 13% in audits 1 and 2, respectively. tPREP consistently increased with the number of operated joints while the time between PREP and surgical incision remained stable (10–15 min). Timings did not improve between the audits (Table 2). In audit 2, tAB-INC was 8 min longer for emergency laparotomy procedures (*p* = 0.03), as a result of a similar increase in tPREP (*p* = 0.04) but the median tAB-INC remained <60 min in both audits (Table 2).

**Table 1:** Surgical preparation phase (tPREP) and antimicrobial dosing to incision time (tAB-INC) for the elective arthroscopy groups for both the audits.

**Number of joints**	**Audit 1**			**Audit 2**		
	**Cases**	**tPREP (min)**	**tAB-INC (min)**	**Cases**	**tPREP (min)**	**tAB-INC (min)**
1	*N* = 25	48 (39–60)	58 (48–73)	*N* = 24	45 (37–67)	60 (50–85)
2	*N* = 15	57 (45–66)	72 (55–85)	*N* = 24	60 (43–78)	72 (52–92)
3	*N* = 5	65 (51–75)	75 (61–88)	*N* = 8	64 (53–80)	79 (64–87)
4 and >4	*N* = 3	83 (63–87)	98 (75–99)	*N* = 4	63 (60–69)	78 (70–81)

*Data are expressed as median (range). The number of joints refers to a single patient and surgical procedure. In the last group (4 and >4), up to six joints were operated*.

**Table 2:** Percentage of surgeries with an antimicrobial dosing to incision time (tAB-INC) <60 min and times [median (range)] for time data.

**Type of surgery/variable**	**Audit 1**	**Audit 2**	***P*-value**	**Median difference**	**95% CI of median difference**
**Elective arthroscopies**
tAB-INC <60 min (%)	37.5 (*n* = 18/48)	23.3 (*n* = 14/60)	0.1	NA	NA
tAB-INC (min)	64.5 (48.0–99.0)	70.0 (50.0–95.0)	0.3	5.5	−2.0–7.0
tPRI (min)	11.5 (4.0– 6.0)	12.5 (6.0–30.0)	0.8	1	−1.0–2.0
tPREP (min)	52.0 (39.0–87.0)	55.0 (37.0–80.0)	0.3	3	−2.0–7.0
**Emergency laparotomies**
tAB-INC <60 min (%)	96 (*n* = 24/25)	83.3 (*n* = 25/30)	0.2	NA	NA
tAB-INC (min)	45.0 (28.0–65.0)	53.0 (22.0–90.0)	0.03^*^	8	0–13.0
tPRI (min)	10.0 (5.0–20.0)	11.0 (0–44.0)	0.5	1	−1.0–3.0
tPREP (min)	34.0 (20.0–52.0)	43.0 (12.0–60.0)	0.04^*^	9	0–11.0

*tPRI, pre-induction phase; tPREP, surgical preparation phase, 95% CI = 95% CI for the median difference. P-values represent comparisons between first and second audits*.

* *Statistical significance*.

## Discussion

In humans, the beneficial effect of timely administration of preoperative antimicrobials to prevent surgical site infections is well-established (16). The same principle is assumed for horses, but the evidence base is weak due to a lack of large-scale, randomized, clinical trials. In contrast to human surgery, antimicrobial prophylaxis is routinely used in clean procedures in horses and this practice has received limited research attention and remains controversial (17). The goal of this study, as with previous work, was to evaluate and improve current practice, based upon the degree of adherence to extrapolated human guidelines (5, 9, 10), and not to assess antimicrobial efficacy. Previous studies reveal a variation in antimicrobial use between institutions as well as differences between intended and actual antimicrobial administration within institutions (5, 9, 10).

In the present study, perioperative antimicrobials were most commonly administered intravenously and primarily consisted of the combination of sodium penicillin and gentamicin. Although no specific studies have been performed to investigate the optimal prophylactic antimicrobials in equine surgery, intravenous penicillin and gentamicin are the most widely used broad-spectrum combination (4). This combination reflects current human guidelines to employ early generation antimicrobials that provide a broad-spectrum coverage against commonly encountered bacteria, and to administer them intravenously (1, 2, 18). In our surgical population, the use of later-generation antimicrobials, such as enrofloxacin and ceftiofur, was generally reserved for the cases where the use of gentamicin was contraindicated due to its nephrotoxic side effects.

The completeness of antimicrobial recording is important for quality of care but also has medico-legal implications (19, 20). Identifying the anesthesia team as specifically responsible for the antimicrobial administration and recording was insufficient to ensure complete recording in all cases in audit 1. This may reflect an unintentional oversight (reliance on memory) or a routine violation of policy. The simple changes introduced before audit 2 (anesthesia record modification and training) were effective in improving compliance for elective arthroscopies but not emergency laparotomies. The arthroscopy results compare favorably with a similar recent study in which 88% of the records were complete (10). In the emergency laparotomy group, an improvement was achieved but this was not statistically significant. Multiple factors may have contributed to the absence of significant improvement, including the smaller sample size (compared to the arthroscopy group, which did show a significant improvement for a similar magnitude of change), miscommunication, the potentially stressful environment surrounding emergency procedures and effects of reduced staffing out of hours. Compliance in anesthesia record keeping has been shown to be poorer in emergency situations in human medicine (21).

In our audits, the tAB-INC fell within the 60 min target for 96% (audit 1) and 83.3% (audit 2) of the emergency laparotomies, with median times falling within the 60-min threshold. This compares favorably with a previous report in which only 11.6% (88/761) of the laparotomies had a tAB-INC <60 min, with a median time of 70 min (5). This disparity may relate to several factors, but the lack of a specific hospital policy regarding responsibility and the timing for antimicrobial prophylaxis could have contributed. For elective arthroscopies, the median tAB-INC achieved in our audits was outside the 60-min threshold (by 5–10 min) and this was associated with the longer tPREP of arthroscopies. However, our results for tAB-INC are approximately half of that previously reported for arthroscopies (142 min) by a different institution, with different hospital policies (9). A retrospective study conducted by the same institution reported an improvement in their rate of arthroscopies achieving a tAB-INC <60 min; improving from 6.3 to 40.2% (10). It is possible that the hospital policy regarding antimicrobial prophylaxis was modified after the first study, but the factors that contributed to this improvement were not described in the second study. In audit 1, 37.5% of the arthroscopies met the <60-min threshold for tAB-INC. However, this percentage increases to 72% if only single joint arthroscopies are considered, which compares favorably to the study by Muntwyler et al. where primarily one-joint arthroscopies (66.3% of cases) were included (10). This also highlights the critical contribution of tPREP to tAB-INC as the number of operated joints increases. In an attempt to shorten tPREP, the presence of two technicians during surgical preparation was encouraged. However, the results of audit 2 did not support our hypothesis. A new surgical technician was being trained during several months of audit 2, and this may have limited achievement in a tPREP reduction. Data specific to the individual were not collected to facilitate further analysis. A potential additional factor to tPREP duration is that horses in our region present with long coats during the winter, extending the clip time and often requiring additional cleaning to remove dirt. In exploratory laparotomies, clipping the surgical site usually begins before anesthesia, a practice that could be considered for arthroscopy procedures in order to shorten tPREP and tAB-INC. However, early surgical site preparation may lead to an increased risk of surgical site infection, though further research is needed to quantify this risk (22, 23). Septic synovitis following arthroscopic surgery has athletic and financial consequences but the specific risks of preoperative clipping have not been investigated (24, 25). Another option to reduce tAB-INC would be the administration of antimicrobials after the induction of anesthesia. There is a general reluctance among anesthesiologists to give antimicrobials, particularly sodium penicillin, during general anesthesia in horses due to a risk of hypotension (26). This warrants further investigation as the incidence of hypotension, severity, and responsiveness to treatment is not well-established. Finally, elective arthroscopies are clean procedures and the need of antimicrobial prophylaxis is controversial. The infection rate after elective arthroscopy remains around 1% irrespective of antimicrobial use (24, 25).

Overall, the change in policy introduced between the two audits was successful in improving antimicrobial recording but not the tAB-INC period. In human medicine, clinical audits introducing protocols or educational programs to improve perioperative antimicrobial prophylaxis had variable success, ranging from 28 to 94.9% for the adherence to newly introduced guidelines (27–30). In contrast to our expectations, a slight increase in the tAB-INC period and the tPREP period was observed for both types of procedures in audit 2. We did not consider the change clinically significant for either group, particularly for emergency laparotomies where tAB-INC remained within 60 min. Nevertheless, the desired improvement in tAB-INC did not occur. Despite the potential effect of training a new surgical technician, it may be that greater education of all surgery team members could result in a shorter tAB-INC. For instance, a greater awareness of the contribution of surgical preparation duration could lead to team members assisting technicians in this phase or being proactive in removing surface dirt before the premedication and antimicrobial administration.

While we anticipate that the general observations made in this study could apply to other hospitals, the current study has several limitations. The findings of this study are limited to the two surgical populations studied, local management practices (the number and degree of training of personnel), and the limited duration of the study. We decided to focus our statistical analysis on the two most commonly performed surgeries to allow the creation of procedurally homogenous groups reflecting different working conditions (emergency/elective). Focusing on these procedures allowed a comparison with the available literature (5, 9, 10). The cutoff value of 60 min was chosen as it is the current guideline for prophylactic antimicrobial use in humans and is also the recommended timeline in the equine literature. The main goal of this guideline is to obtain tissue and plasma antimicrobial concentrations that exceed the minimal inhibitory concentration for most organisms likely to be encountered at the time of surgical incision. While the time to achieve appropriate tissue and plasma concentrations would be expected to vary according to the pharmacokinetic properties of the selected antimicrobial, studies analyzing the risk of surgical site infections continue to find a 60 min cutoff value to be a useful indicator for when the risk of infection increases (31).

The planned study ended with the “Study” phase of audit 2. Further audit cycles are needed to establish the longevity of observed improvements and achieve further improvement. Our assessment was also limited to the recording and timing of antimicrobials while other factors such as initial dosing, intraoperative redosing, and the rate of surgical site infection were not considered in our study, though they play an essential role in the efficacy of perioperative antimicrobial prophylaxis.

In conclusion, achieving the recommended timing of perioperative antimicrobials remains challenging in equine elective arthroscopic procedures and clinical audits are useful in identifying a deviation from best practices and the effect of introduced changes. We encourage other institutions to audit their perioperative use of antimicrobials with the goal of documenting adherence to the current extrapolated guidelines and build the literature necessary to introduce species-specific evidence-based guidelines.

## Data Availability Statement

The datasets presented in this study can be found in online repositories. The names of the repository/repositories and accession number(s) can be found at: https://doi.org/10.7910/DVN/FAPILE.

## Author Contributions

AB and DP conceived the idea of the work. SC, AB, and DP designed the study. SC and RW collected the data. SC and DP performed statistical analysis. All authors prepared and edited the manuscript prior to submission.

## Conflict of Interest

The authors declare that the research was conducted in the absence of any commercial or financial relationships that could be construed as a potential conflict of interest.
